# Genome-Wide Identification and Expression Analysis of the *CaM* Gene Family in Tree Peony (*Paeonia ostii*) During the Pistil Pollination Process

**DOI:** 10.3390/cimb47100816

**Published:** 2025-10-02

**Authors:** Guodong Zhao, Shuran Lv, Yuxin Zhao, Yuying Li, Xiaogai Hou

**Affiliations:** College of Agriculture/Tree Peony, Henan University of Science and Technology, Luoyang 471023, China; zhaoguodong2025@163.com (G.Z.); lvshuranll@163.com (S.L.); 17329296406@163.com (Y.Z.); hkdhxg@haust.edu.cn (X.H.)

**Keywords:** *Paeonia ostii* ‘Fengdan’, Calmodulin (CaMs), gene family, expression analysis

## Abstract

Tree peony is an important horticultural plant with both ornamental and oil value. The tree peony genome and databases were used to search for calmodulin family genes to explore their function in the pollination of tree peony. The CaM gene family was identified, and then the basic protein characteristics of the family members, such as gene structure, isoelectric point, molecular weight, subcellular localization, and conserved protein domain, were analyzed. The expression levels of these genes in the pistil tissue of *Paeonia ostii* ‘Fengdan’ at different developmental stages after pollination were also analyzed. Further, qRT-PCR was used to detect the expression levels of six *PsCaMs* during the development process of the pistil under bee pollination conditions. The results showed that there were six CaM family members located on three chromosomes and one non-chromosome. There were a large number of hormone response and stress response elements on the gene promoter of this family. During the development of pistil tissue after pollination, CaM family gene expression showed a trend of first increasing and then decreasing, which may be related to its function during pollination. The purpose of this study is to identify the gene characteristics and expression patterns of the CaM family during pollination, and to lay a foundation for the functional study of the CaM family in tree peony pollination.

## 1. Introduction

*Paeonia* section *Moutan* DC., a woody plant belonging to the family Paeoniaceae, is widely recognized for its large, full blossoms, diverse flower forms, vibrant colors, and intense fragrance. It is revered as the “King of Flowers” and a symbol of wealth and prosperity, with significant ornamental value [1]. In addition to its aesthetic appeal, tree peony has emerged as a novel woody oil crop with substantial economic potential [2]. The seed oil of tree peony contains up to 92.26% unsaturated fatty acids, including over 42% α-linolenic acid. Therefore, the exploration and utilization of *Paeonia* germplasm rich in α-linolenic acid are of great application value [3].

Most species within the *Paeonia* section *Moutan* exhibit self-incompatibility [4]. This is particularly pronounced in *Paeonia ostii* ‘Fengdan’, where the self-pollination seed set rate is extremely low, with an average of only 0.4 seeds per flower [5]. Bee pollination has been shown to significantly improve seed yield and mitigate the adverse effects of late spring frost on seed development [6]. Studies have indicated that pollination is influenced by various chemical factors. For example, pollen germination increases with sucrose concentrations up to 150 g·L^−1^. Moreover, 80 mg·L^−1^ boric acid significantly enhances pollen germination [7]. Boric acid facilitates Ca^2+^ influx by interacting with calcium channels on the plasma membrane, triggering intracellular calcium mobilization and opening internal Ca^2+^ stores, thereby elevating cytosolic Ca^2+^ levels [8].

Calcium (Ca^2+^) plays a crucial role in pollen tube growth. At low Ca^2+^ concentrations, pollen tube elongation slows or ceases, whereas excessive Ca^2+^ levels also inhibit growth [9]. Prior to pollen tube germination, Ca^2+^ accumulates near the germination pore. Following germination, a calcium gradient forms along the pollen tube, regulating vesicle trafficking, fusion, and secretion from the Golgi apparatus. This ensures a continuous supply of cell wall and plasma membrane materials to the tube tip, supporting its polarized growth [10].

Calmodulin (CaM) proteins, ubiquitous in plants, are encoded by multigene families producing identical or highly similar proteins [11]. CaMs are central regulators in a wide range of physiological processes, including cell morphogenesis, division, flowering, autophagy, protoplast wall regeneration, and pollen tube germination [12]. Additionally, CaMs are involved in responses to abiotic stresses such as mechanical stimulation, drought, chilling [13], heat, and high salinity [14]. With the advent of whole-genome sequencing, the CaM gene family has been identified and characterized in many plant species. In *Arabidopsis*, *AtCaM4* interacts with *PALT1* to repress *CBF* gene expression, negatively regulating cold tolerance [15], while *AtCaM2* regulates pollen tube orientation and signaling by modulating calcium levels during growth [16]. In maize, CaM promotes pollen germination and tube elongation, whereas CaM inhibitors such as *CPZ*, *W7*, *TFP*, and compound 48/80 strongly inhibit these processes [17]. In *Cucumis sativus*, *CsCaM3* enhances thermotolerance by upregulating *HSP70* and *HSP90* expression and alleviating membrane damage [18]. In apple, *MdCaM2* interacts with *MdCRF4* in a Ca^2+^-dependent manner, phosphorylating and suppressing *MdCRF*4-induced *MdACS1* transcription, thereby reducing ethylene biosynthesis and delaying fruit ripening [19]. In rice, *OsCaM* interacts with *OsCNGC13*, a channel critical for transmitting pollen through the style; loss of *OsCNGC13* impairs pollen transport to the ovary [20]. These findings demonstrate that CaMs participate in complex calcium-mediated signaling pathways, playing indispensable roles in plant development, stress responses, and hormone signaling.

In this study, pistil tissues of *Paeonia ostii* ‘Fengdan’ at different developmental stages after bee pollination were used as experimental materials. Based on the *Paeonia ostii* genome and other public databases, we identified CaM gene family members in tree peony, analyzed their physicochemical properties, conserved motifs, phylogenetic relationships, chromosomal locations, and expression patterns. This work lays a foundation for further investigation into the molecular mechanisms underlying pollination in tree peony.

## 2. Materials and Methods

### 2.1. Plant Materials

Experimental materials were collected from the Tree Peony Experimental Base of Henan University of Science and Technology (112°25′16.28″ E, 34°36′8.00″ N). Twelve-year-old healthy *Paeonia ostii* ‘Fengdan’ plants with consistent growth were selected. Bee pollination was performed prior to flowering in April 2023. Pistil tissues of *Paeonia ostii* ‘Fengdan’ were harvested at 0 h (non-pollinated), 1 h, and 2 h after pollination. Five pistil tissues from each plant were collected, and the samples were immediately frozen in liquid nitrogen and stored at –80 °C for subsequent analysis.

### 2.2. Experimental Methods

#### 2.2.1. Identification of CaM Gene Family Members and Protein Characterization in Tree Peony

Seven annotated *Arabidopsis thaliana CaM* protein sequences were downloaded from the TAIR database (http://www.arabidopsis.org/, accessed on 20 April 2024). These sequences were used as queries for BLAST searches against the *Paeonia* genome [21] using NCBI-BLASTp (https://blast.ncbi.nlm.nih.gov/Blast.cgi, accessed on 20 April 2024). Candidate homologous sequences with >50% similarity was retained. After removing redundant sequences, putative CaM family members in *Paeonia* were identified.

The physicochemical properties of PsCaM proteins, including isoelectric point, molecular weight, and conserved domains, were predicted using ExPASy (https://web.expasy.org), SMART (http://smart.embl-heidelberg.de/), Pfam (http://pfam.xfam.org/), and TBtools software (v. 2.031). Subcellular localization was predicted using Cell-Ploc 2.0 (http://www.csbio.sjtu.edu.cn/bioinf/Cell-PLoc-2/, accessed on 25 May 2024), BUSCA (http://busca.biocomp.unibo.it), and YLoc (https://github.com/KohlbacherLab/YLoc, accessed on 25 May 2024). Chromosomal localization was visualized using TBtools. All the above-mentioned online websites and software use default parameters.

#### 2.2.2. Phylogenetic Analysis of CaM Gene Family Members

Multiple sequence alignments of CaM proteins from *Paeonia* and *Arabidopsis* were performed using ClustalX (www.clustal.org, accessed on 16 June 2024). A phylogenetic tree was constructed using the Neighbor-Joining (NJ) method implemented in MEGA software (www.megasoftware.net, accessed on 19 June 2024). The program parameters were set as follows: Bootstrap replication times were 1000, Poisson model, complete state deletion, and other parameters were set to default.

#### 2.2.3. Conserved Motif and Gene Structure Analysis of CaM Genes

The conserved motifs of PsCaM proteins were identified using MEME (https://meme-suite.org) based on the expectation-maximization (EM) algorithm. The results were visualized using TBtools. Gene structures and cis-acting elements located in the 2000 bp upstream promoter regions of candidate genes were also analyzed using TBtools (v. 2.031), and key elements were selected for further visualization.

#### 2.2.4. Expression Analysis of PsCaM Genes Based on RNA-Seq Data

Based on previously obtained RNA-seq data from pistil tissues of *P. ostii* ‘Fengdan’ under bee and self-pollination conditions, the time expression patterns of *PsCaM* genes were analyzed during pistil development.

##### 2.2.5. qRT-PCR Validation of PsCaM Gene Expression

Total RNA was extracted from pistil tissues of *P. ostii* ‘Fengdan’ at 0, 1, and 2 h after bee pollination, with 2 g used for each sample, using the RNAprep Pure Plant Kit for polysaccharide- and polyphenol-rich tissues (DP441, TIANGEN Biotech, Beijing, China). RNA concentration and quality were assessed using 1.0% agarose gel electrophoresis and a NanoDrop spectrophotometer.

qRT-PCR was performed using the Evo M-MLV Reverse Transcription Kit (AG11705, Accurate Biology, Hunan, China) and SYBR Green Pro Taq HS Premix Kit (AG11701, Accurate Biology, Hunan, China) on the BIO-RAD CFX Connect Real-Time PCR Detection System. Each sample was subjected to a reverse transcription reaction using 100 ng of RNA. Gene-specific primers for *PsCaMs* were designed using Primer Premier (Table 1), with *PsEF-1α* as the internal reference gene, and the final volume of the qRT-PCR reaction was 20 μL [22]. Three biological replicates were included for each sample. Relative gene expression levels were calculated using the 2^−ΔΔCt^ method, and statistical significance was determined via one-way analysis of variance (ANOVA).

## 3. Results

### 3.1. Identification and Physicochemical Properties of CaM Gene Family Members in Paeonia suffruticosa

A total of six CaM family members were identified from the *P. ostii* genome (Table 2). Based on sequence homology with *A. thaliana* CaMs, these genes were designated as *PsCaM1* to *PsCaM6*. The amino acid lengths of PsCaMs ranged from 450 to 2217, with PsCaM4 being the longest. The average molecular weight was approximately 63,719.06 ± 24,269.72 Da, and the average isoelectric point (pI) was 5.21 ± 0.05. The instability indices ranged from 19.16 to 41.17, the aliphatic indices from 29.11 to 36.20, and the grand average of hydropathicity (GRAVY) values fell between 0.67 and 0.75, indicating that all PsCaMs are hydrophobic proteins. Subcellular localization predictions suggested that five of the six PsCaMs were primarily localized to the plasma membrane, with PsCaM6 uniquely localized to the vacuole.

### 3.2. Chromosomal Localization of PsCaM Genes

**Figure 1:**
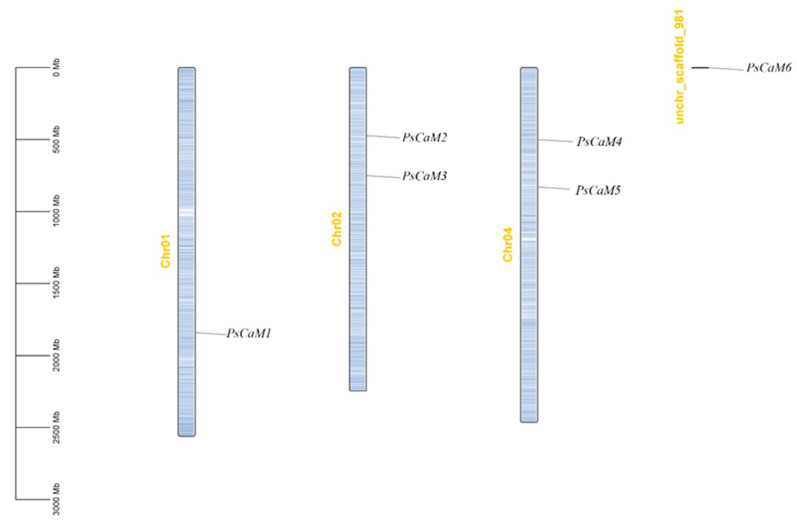
Chromosomal localization of the PsCaM family members in tree peony.

*Paeonia ostii* has relatively few chromosomes (2n = 10) but large chromosome sizes. The six *PsCaM* genes were distributed unevenly across three chromosomes: Chr1, Chr2, and Chr4 (Figure 1). Specifically, *PsCaM*1 was located on Chr1; *PsCaM*2 and *PsCaM*3 on Chr2; and *PsCaM*4 and *PsCaM*5 on Chr4. *PsCaM*6 was not anchored to any chromosome in the current genome assembly.

### 3.3. Phylogenetic Analysis of CaM Family Members

**Figure 2:**
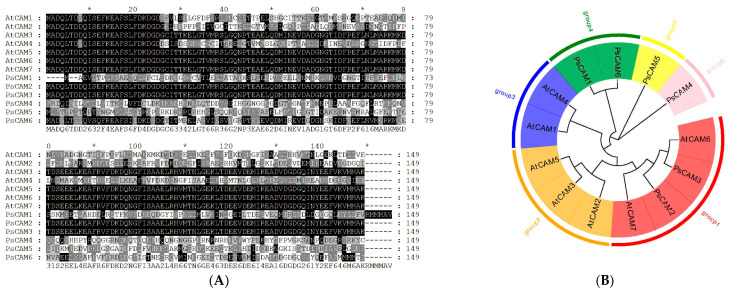
Amino acid sequence alignment and phylogenetic tree of CaMs in tree peony and other species. (**A**) Multiple sequence alignment of CaM protein from tree peony and Arabidopsis. (**B**) Phylogenetic tree of CaM protein from tree peony and Arabidopsis.

Multiple sequence alignment revealed high conservation among the 13 CaM proteins from *Paeonia ostii* and *A. thaliana* (Figure 2A). These CaMs were classified into six groups. The six PsCaMs were distributed across groups 1, 4, 5, and 6, containing 2, 2, 1, and 1 members, respectively. PsCaM2 and PsCaM3 showed high sequence similarity with AtCaM6 and AtCaM7; PsCaM1 and PsCaM6 were more closely related to AtCaM1 and AtCaM4. In contrast, PsCaM4 and PsCaM5 showed lower homology with other CaM proteins (Figure 2B).

### 3.4. Conserved Motif and Gene Structure Analysis of PsCaMs

**Figure 3:**
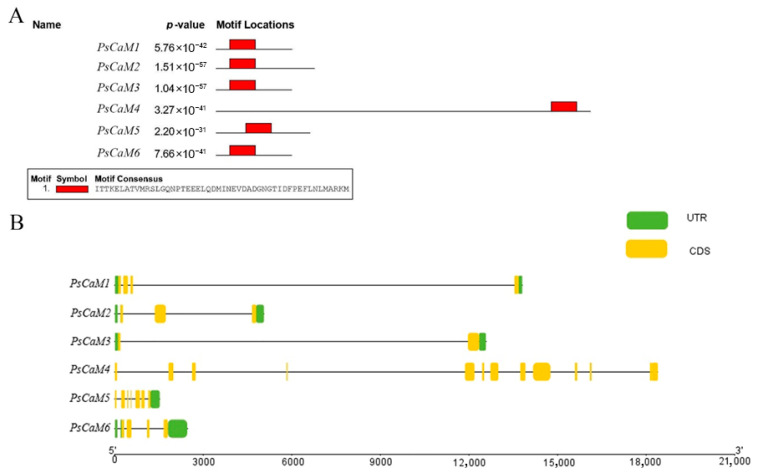
Conserved motifs and gene structure of the *PsCaM* family members. (**A**) Conserved motifs identified in members of the PsCaM family are represented by red boxes. (**B**) Gene structure showing the exon-intron organization, where the UTRs and CDS are represented as green and yellow boxes, respectively. Introns are represented as lines.

Motif analysis using MEME revealed that all six *PsCaMs* shared a highly conserved motif with the amino acid sequence: ITTKELATVMRSLGQNPTEEELQDMINEVDADGNGTIDFPEFLNLMARKM with a maximum *p*-value of 2.20 × 10^–31^ (Figure 3A), confirming their identity as CaM family members. The motifs in PsCaM1, PsCaM2, PsCaM3, PsCaM5, and PsCaM6 were mainly located in the central regions of the protein sequences.

Gene structure analysis based on genome annotation showed that PsCaM4 had the longest gene length (~18,000 bp) and included 13 CDS regions, though lacking both 5′UTR and 3′UTR. PsCaM5 was the shortest (~1500 bp), containing 7 CDS regions and a 5′UTR but lacking a 3′UTR. The remaining members contained 2–4 CDS regions and both UTRs (Figure 3B).

### 3.5. Cis-Regulatory Elements in Promoters of PsCaMs

Cis-element analysis of the 2000 bp upstream promoter regions revealed 11–19 regulatory elements per *PsCaM* gene, including hormone-responsive (ABRE), low-temperature responsive (LTR), light-responsive (G-box), drought-inducible (MBS), and anaerobic-inducible (ARE) elements (Table 3). All *PsCaMs* possessed abundant light- and hormone-responsive elements. Only *PsCaM3* and *PsCaM5* had drought-responsive elements, suggesting that most *PsCaMs* may respond to phytohormone signaling and light stimuli.

### 3.6. Expression Patterns of PsCaM Family Genes During Pistil Development Following Pollination in P. ostii ‘Fengdan’

**Figure 4:**
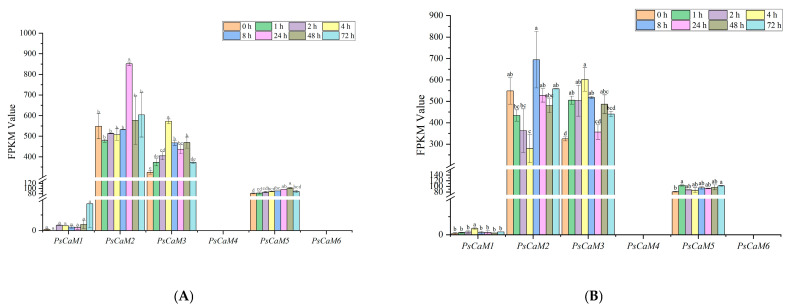
Expression analysis of the PsCaM family members in the pistils of tree peony at different developmental stages based on RNA-seq data. (**A**) Expression analysis of *PsCaM* family members at different developmental stages of peony pistil after bee pollination. (**B**) Expression analysis of *PsCaM* family members at different developmental stages of peony pistil after self-pollination. Lowercase letters a-e indicate significance at *p* < 0.05.

**Figure 5:**
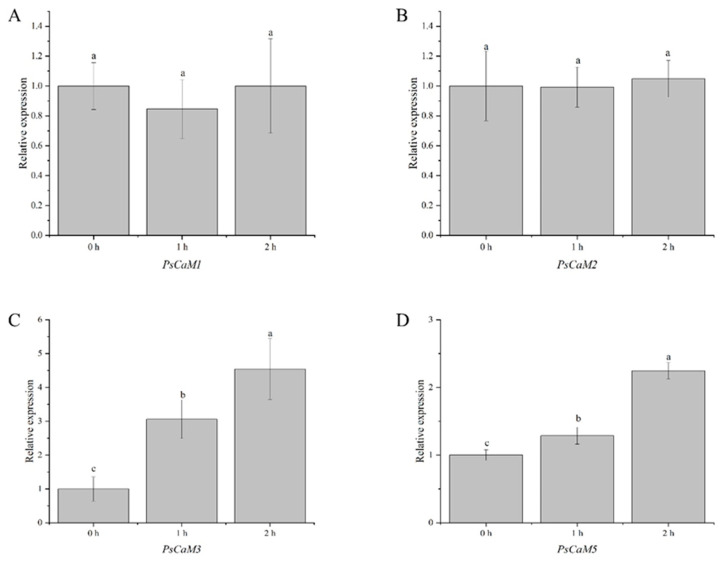
qRT-PCR detection of the PsCaM family genes under bee pollination conditions. (**A**) *PsCaM1*, (**B**) *PsCaM2*, (**C**) *PsCaM3*, (**D**) *PsCaM5*. Lowercase letters a-c indicate significance at *p* < 0.05.

RNA-seq data from previous experiments indicated that, except for *PsCaM4* and *PsCaM6*, the remaining *PsCaMs* were expressed in pistil tissues under both bee and hand pollination. Expression levels varied among members (Figure 4). *PsCaM2* and *PsCaM3* exhibited higher expression across developmental stages. *PsCaM2* showed a dynamic pattern of initial downregulation followed by a sharp increase and subsequent decline, peaking at 24 h post-bee pollination (*p* < 0.05; Figure 4A) and at 8 h under hand pollination (*p* < 0.05; Figure 5B). *PsCaM3* showed a peak expression at 4 h post-pollination under both conditions (*p* < 0.05; Figure 4A,B).

qRT-PCR analysis validated the expression of *PsCaMs* at 0, 1, and 2 h post bee pollination (Figure 5), consistent with RNA-seq results. *PsCaM1* and *PsCaM2* exhibited an initial decrease followed by an increase, but changes were not statistically significant (*p* > 0.05; Figure 5A,B). In contrast, *PsCaM3* and *PsCaM5* showed significantly increased expression, peaking at 2 h post-pollination (*p* < 0.05; Figure 5C,D), suggesting a potential shared regulatory function. *PsCaM4* and *PsCaM6* showed no detectable expression at any time point post-pollination.

## 4. Discussion

Calmodulin (CaM) is an acidic protein composed of 148 amino acids, with a molecular mass ranging from 16.7 to 16.8 kDa and a pI of approximately 4.0 [23]. It lacks easily oxidizable residues such as cysteine and tryptophan, which confer high thermal stability, even retaining bioactivity at 90 °C. CaMs were highly conserved across eukaryotes, with up to 91% sequence similarity between plant and vertebrate CaMs, 61% similarity with yeast, and 84–100% similarity among plants and algae [24]. Structurally, CaM contains four Ca^2+^-binding EF-hand motifs, seven α-helices, and two antiparallel β-sheets [25]. The presence of only four EF-hand domains and no additional functional motifs is a defining feature of the CaM gene family [26]. Although CaM genes have been studied in many species, little is known about their function and number in tree peony. This study analyzed the sequence characteristics of CaM gene members and their expression patterns during pollination of tree peony, which laid a foundation for the study of the CaM gene in tree peony.

CaMs were widely distributed in various species. In *A. thaliana*, seven CaM genes encode four isoforms differing by only 1–5 amino acids [27]. In rice, five genes encode three isoforms with >97% similarity [28]. And in wheat, 40 genes encode 13 highly similar isoforms [29]. Similarly, three CaM genes in grape belong to a single subfamily, and analogous CaM gene families have also been reported in soybean, tobacco, and maize [30,31,32,33]. In this study, six *PsCaMs* were identified from the *P. ostii* ‘Fengdan’, of which *PsCaM1*, *PsCaM2*, *PsCaM3*, and *PsCaM6* exhibited high sequence conservation. We performed multiple sequence alignments between PsCaM and AtCaM, and the results showed that PsCaM7 had the closest homology with AtCaM2. Among them, *PsCaM4* showed moderate similarity, while *PsCaM5* was more divergent. All six contained four canonical EF-hand motifs without additional conserved domains and were primarily localized to the cytoplasm or plasma membrane, which is consistent with the characteristics of the typical calmodulin family [14]. These findings lay the groundwork for future functional investigations of CaMs in *Paeonia ostii* ‘Fengdan’.

CaM gene expression varies among developmental stages and tissues. In *Pyrus bretschneideri*, *CaMs* were differentially expressed in young leaves, mature leaves, flower buds, and petals, which is likely to reflect the diversity of their functions [34]. In fig (*Ficus carica*), five CaM genes show distinct responses to cold, light, drought, and ABA treatments, suggesting coordinated regulation among family members under stress conditions [35]. CaM was also implicated in multiple aspects of pollen germination. Although *CaMs* were ubiquitous in the cytoplasm, their activation strictly depends on Ca^2+^ binding, leading to localized activation in regions with high Ca^2+^ gradients, such as pollen tube tips, where active CaM can negatively regulate targets like cyclic nucleotide-gated channels (CNGCs) [36]. Furthermore, CaM interacts with Ca^2+^-dependent kinases to regulate pollen tube growth and directional guidance [37]. In this study, under bee pollination, *PsCaM1* and *PsCaM2* showed transient downregulation followed by upregulation in pistil tissues, whereas *PsCaM3* and *PsCaM5* were significantly upregulated post-pollination, likely reflecting roles in post-transcriptional or translational regulatory networks, warranting further investigation [38]. We analyzed the expression pattern of the CaM gene in different stamen development stages of tree peony under bee pollination and self-pollination conditions. It was found that the expression patterns of CaM2 and CaM3 in self-pollinated and honey-pollinated tree peony were slightly different, suggesting that they may be involved in the regulation of the tree peony bee pollination process.

Hormone signals also play a significant role in pollen germination and pollen tube growth, and there is a close interaction between calcium signals and hormone signals. Current data indicate that pollen germination, both in vitro and in vivo, is accompanied by endogenous plant hormones. The identification of CaM family members in peonies has revealed 2–7 hormone responses, suggesting that *PsCaMs* may be involved in the hormone-induced response process of tree peonies, thereby facilitating the participation of calcium signals in regulating aspects such as pollen germination and pollen tube elongation to regulate the growth and development of plants. This provides clues for further in-depth molecular mechanism research on these genes in the future.

## 5. Conclusions

Six *PsCaMs* were identified and obtained from the genome of the tree peony. Each member was unevenly located on three chromosomes. A large number of hormone response and stress response elements were present in the promoters of these six *PsCaMs*. During the development process of the pistil tissue of *Paeonia ostii* ‘Fengdan’ after pollination, the expression levels of CaM family genes showed a trend of first increasing and then decreasing, which may be related to their functions during the pollination process.

## Figures and Tables

**Table 1:** The information on qRT-PCR primers.

Primer Name	Forward Primer	Reverse Primer
*PsCaM1*	5′-GATGGAGATGGCTGCGTTAC-3′	5′-CAACTCGTCGTGTGCTTCAG-3′
*PsCaM2*	5′-GGTTGATGCTGATGGGAATG-3′	5′-TGAAGCCGTTTTGGTCCT-3′
*PsCaM3*	5′-CGGTTGTATCACCACGAAAG-3′	5′-ATTCTTCCTCAGAGTCGGTGTC-3′
*PsCaM4*	5′-GTTTCTGGGATTCACGGAGG-3′	5′-GGACCACCATTACCGTTCTG-3′
*PsCaM5*	5′-AACACAGCCTACAGCCAATG-3′	5′-TGTCATCTCAAAGCCCAGTG-3′
*PsCaM6*	5′-GAAGAACTGGCAGCGGTCAT-3′	5′-CCTCTGCGACATTTTCCTGC-3′
*EF-1α*	5′-CCGCCAGAGAGGCTGCTAAT-3′	5′-GCAATGTGGGAAGTGTGGCA-3′

**Table 2:** Physicochemical properties and subcellular localization of CaM family members in tree peony.

Gene ID	Number of Amino Acids	Molecular Weight/kD	pI	Instability Index	Aliphatic Index	Grand Average of Hydropathicity	Subcellular Localization
*PsCaM1*	453	35,954.28	5.30	31.79	36.20	0.72	Cytomembrane, cytoplasm
*PsCaM2*	585	46,161.92	5.21	40.72	31.11	0.75	Cytomembrane, cytoplasm
*PsCaM3*	450	36,013.58	5.26	34.73	29.11	0.67	Cytomembrane, cytoplasm
*PsCaM4*	2217	184,707.70	4.95	41.17	32.48	0.73	Cytomembrane
*PsCaM5*	558	43,948.14	5.25	35.04	35.30	0.74	Cytomembrane
*PsCaM6*	450	35,528.73	5.30	19.16	33.78	0.68	Vacuole

**Table 3:** Analysis of cis-acting elements of the PsCaM family promoter.

Genes	ABRE	MBS	LTR	ARE	G-Box
*PsCaM1*	7	0	0	2	10
*PsCaM2*	2	0	0	2	11
*PsCaM3*	2	1	0	2	10
*PsCaM4*	2	0	1	1	7
*PsCaM3*	2	1	0	6	2
*PsCaM6*	2	0	1	1	12

## Data Availability

The original contributions presented in this study are included in the article. Further inquiries can be directed to the corresponding authors.

## References

[1] Li Y.Y., Guo L.L., Wang Z.Y., Zhao D.H., Guo D.L., Carlson J.E., Yin W.L., Hou X.G. (2023). Genome-wide association study of twenty-three flowering phenology traits and four floral agronomic traits in tree peony (*Paeonia* section *Moutan* DC.) reveals five genes known to regulate flowering time. Hortic. Res..

[2] Li Y.Y., Guo Q., Zhang K.Y., Wang H., Jia C.S., Guo D.L., Guo L.L., Hou X.G. (2022). Dormancy-release, germination and seedling growth of Paeonia ostii ‘Fengdan’ seeds under measures of physical and chemical treatment and sowing. PLoS ONE.

[3] Zhang K.Y., Wang X., Bao J.Y., He X.N., Leri Y., He C.L., Hou X.G. (2024). Bumblebee pollination ensures the stability of both yield and quality of the woody oil crop *Paeonia ostii* ‘Fengdan’. Basic Appl. Ecol..

[4] Zhang K.L., Yao L.J., Zhang Y., Baskin J.M., Baskin C.C., Xiong Z.M., Tao J. (2019). A review of the seed biology of *Paeonia* species (Paeoniaceae), with particular reference to dormancy and germination. Planta.

[5] Wang H., Wei S., He Y.L., Wang X.H., Li Y.Y., Wei D.F., Wang Z.Y., Guo L.L., Shaaban M., Hou X.G. (2023). Characterization of agronomic and seed oil features for different cultivars of tree peony. Plants.

[6] Zhang K.Y., He C.L., Hou X.G. (2022). Influence of pollination methods on fruit development, fruit yield and oil quality in oil tree peony. Sci. Hortic..

[7] Shi J., Wang X.Y., Zhang S.L., Huo Z.P., Liu S.D. (2013). The influence of culture medium components on the germination of peony pollen and the growth of pollen tubes. J. Henan Univ. Sci. Technol. (Nat. Sci. Ed.).

[8] Wang H., Zhang C.H., Huang Y.L. (2023). Research progress on factors affecting plant pollen and preservation methods. Chin. Agric. Sci. Bull..

[9] Luo J.Y., Tao Q., Wang Y., Pei S.Y., Zou X.X., Yuan F. (2025). The role of calcium signaling in pollen germination and pollen tube growth. Trop. Crops J..

[10] Zhou Z.G., Zheng S., UI Haq S.I., Zhang D.F., Qiu Q.S. (2022). Regulation of pollen tube growth by cellular pH and ions. J. Plant Physiol..

[11] Zuo N., Chen J., Lv Y.G. (2016). Advance progress in plant calmodulin and calmodulin-binding proteins structure biology. Cereals Oils.

[12] Gong M., Cao Z.X. (1995). Regulation of calcium and calmodulin on pollen germination and growth of pollen tube. Plant Physiol. Commun..

[13] Zhang W., Zhou R.G., Gao Y.J., Zheng S.Z., Xu P., Zhang S.Q., Sun D.Y. (2009). Molecular and genetic evidence for the key role of *AtCaM3* in heat-shock signal transduction in *Arabidopsis*. Plant Physiol..

[14] Liu S., Zhao L., Cheng M., Sun J.F., Ji X.M., Ullah A., Xie G.S. (2024). Calmodulins and calmodulin-like proteins-mediated plant organellar calcium signaling networks under abiotic stress. Crop J..

[15] Chu M.X., Li J.J., Zhang J.Y., Shen S.F., Li C.N., Gao Y.J., Zhang S.Q. (2018). *AtCaM4* interacts with a Sec14-like protein, PATL1, to regulate freezing tolerance in *Arabidopsis* in a CBF-independent manner. J. Exp. Bot..

[16] Yu X.J., Cao S.Y., Dong Y.M., Bi B.L., Zhang Y.H., Xu J.Q. (2016). Research progress on the regulation of pollen growth and development by calcium-binding proteins. Northwest Bot. J..

[17] Duan H.S., Wang C.T., Wei F.J. (2023). Identification and characterization of the CaM gene family in maize. Cereal Sci..

[18] Yu B.W., Yan S.S., Zhou H.Y., Dong R.Y., Lei J.J., Chen C.M., Cao. B.H. (2018). Overexpression of *CsCaM3* improves high temperature tolerance in cucumber. Front. Plant Sci..

[19] Li T., Liu Z., Lv T.X., Xu Y.X., Wei Y., Liu W.T., Wei Y.J., Liu L., Wang A.D. (2023). Phosphorylation of *MdCYTOKININ RESPONSE FACTOR4* suppresses ethylene biosynthesis during apple fruit ripening. Plant Physiol..

[20] Xu Y., Yang J., Wang Y.H., Wang J.C., Yu Y., Long Y., Wang Y.L., Zhang H., Ren Y.L., Chen J. (2017). *OsCNGC13* promotes seed-setting rate by facilitating pollen tube growth in stylar tissues. PLos Genet..

[21] Yuan J.H., Jiang S.J., Jian J.B., Liu M.Y., Yue Z., Xu J.B., Li J., Xu C.Y., Lin L.H., Jing Y. (2022). Genomic basis of the giga-chromosomes and giga-genome of tree peony *Paeonia ostii*. Nat. Commun..

[22] Li Y.Y., Wang C., Guo Q., Song C.W., Wang X.H., Guo L.L., Hou X.G. (2022). Characteristics of *PoVIN3*, a key gene of vernalization pathway, affects flowering time. Int. J. Mol. Sci..

[23] Wang H.B., Feng M.C., Zhong X.Q., Yu Q., Que Y.X., Xu L.P., Guo J.L. (2022). Identification of saccharum CaM gene family and function characterization of *ScCaM1* during cold and oxidant exposure in *Pichia pastoris*. Genes Genom..

[24] Zielinski R.E. (1998). Calmodulin and calmodulin-binding proteins in plants. Annu. Rev. Plant Physiol. Plant Mol. Biol..

[25] Minnes L., Shaw D.J., Cossins B.P., Donaldson P.M., Greetham G.M., Towrie N., Parker A.W., Baker M.G., Henry A.J., Taylor R.J. (2017). Quantifying Secondary Structure Changes in Calmodulin Using 2D-IR Spectroscopy. Anal. Chem..

[26] Gao L. (2023). Systematic Evolution of Plant CaM/CML Gene Family and Its Structural and Expression Characteristics in Lotus. Master’s Thesis.

[27] Mccormack E., Braam J. (2003). Calmodulins and related potential calcium sensors of *Arabidopsis*. New Phytol..

[28] Boonburapong B., Buaboocha T. (2007). Genome-wide identification and analyses of the rice calmodulin and related potential calcium sensor proteins. BMC Plant Biol..

[29] Liu Y.W., Chen W.Y., Liu L.B., Su Y.H., Li Y., Jia W.Z., Jiao B., Wang J., Yang F., Dong F.S. (2022). Genome-wide identification and expression analysis of calmodulin and calmodulin-like genes in wheat (*Triticum aestivum* L.). Plant Signal. Behav..

[30] Vandelle E., Vannozzi A., Wong D., Danzi D., Digby A.M., Santo S.D., Astegno A. (2018). Identification, characterization, and expression analysis of calmodulin and calmodulin-like genes in grapevine reveal likely roles in stress responses. Plant Physiol. Biochem..

[31] Crego C.G., Hess J., Yardeni G., Harpe M.A., Priemer C., Beclin F., Saadain S., Cauz-Santos L.A., Temsch E.M., Weiss-Schneeweiss H. (2024). CAM evolution is associated with gene family expansion in an explosive bromeliad radiation. Plant Cell.

[32] Li Q.H., Gao L., Yu F., Lv S.Y., Yang P.F. (2023). Evolution and diversification of CaM/CML gene family in green plants. Plant Physiol. Biochem..

[33] Zhao Y., Liu W., Xu Y.P., Cao J.Y., Braam J., Cai X.Z. (2013). Genome-wide identification and functional analyses of calmodulin genes in solanaceous species. BMC Plant Biol..

[34] Cheng Y.D., Ge W.Y., Yan H.B., Yang K., Guan J.F. (2016). Cloning and expression analysis of calmodulin gene from “Yali” pears. J. Agric. Univ. Hebei.

[35] Nie F., Fang H.L., Liu H.P., Qi X.W., Yu F., Li L., Liang C.Y. (2024). Cloning and expression analysis on *FcCaM* gene from *Ficus carica*. J. Plant Resour. Environ..

[36] Zhou L., Fu Y., Yang Z. (2009). A genome-wide functional characterization of *Arabidopsis* regulatory calcium sensors in pollen tubes. J. Integr. Plant Biol..

[37] Gao Q., Wang C., Xi Y., Shao Q.L., Li L.G., Luan S. (2022). A receptor-channel trio conducts Ca^2+^ signalling for pollen tube reception. Nature.

[38] El Mahi H., Pérez-Hormaeche J., De Luca A., Villalta I., Espartero J., Gámez-Arjona F., Fernández J.L., Bundó M., Mendoza I., Mieulet D. (2019). A critical role of sodium flux via the plasma membrane Na^+^/H^+^ exchanger SOS_1_ in the salt tolerance of rice. Plant Physiol..

